# A microscopy-based small molecule screen in primary neurons reveals neuroprotective properties of the FDA-approved anti-viral drug Elvitegravir

**DOI:** 10.1186/s13041-020-00641-1

**Published:** 2020-09-14

**Authors:** Simon F. Merz, C. Peter Bengtson, Clara Tepohl, Anna M. Hagenston, Hilmar Bading, Carlos Bas-Orth

**Affiliations:** 1 Department of Neurobiology, Interdisciplinary Center for Neurosciences, Heidelberg University, Im Neuenheimer Feld 366, D-69120 Heidelberg, Germany; 2 Department of Medical Cell Biology, Institute for Anatomy and Cell Biology, Heidelberg University, Im Neuenheimer Feld 307, D-69120 Heidelberg, Germany

**Keywords:** Excitotoxicity, Mitochondria, Neuroprotection, NMDA receptor, Glutamate toxicity, Dutasteride, Finasteride, Oxybutynin, Channelrhodopsin

## Abstract

Glutamate toxicity is a pathomechanism that contributes to neuronal cell death in a wide range of acute and chronic neurodegenerative and neuroinflammatory diseases. Activation of the *N*-methyl-D-aspartate (NMDA)-type glutamate receptor and breakdown of the mitochondrial membrane potential are key events during glutamate toxicity. Due to its manifold functions in nervous system physiology, however, the NMDA receptor is not well suited as a drug target. To identify novel compounds that act downstream of toxic NMDA receptor signaling and can protect mitochondria from glutamate toxicity, we developed a cell viability screening assay in primary mouse cortical neurons. In a proof-of-principle screen we tested 146 natural products and 424 FDA-approved drugs for their ability to protect neurons against NMDA-induced cell death. We confirmed several known neuroprotective drugs that include Dutasteride, Enalapril, Finasteride, Haloperidol, and Oxybutynin, and we identified neuroprotective properties of Elvitegravir. Using live imaging of tetramethylrhodamine ethyl ester-labelled primary cortical neurons, we found that Elvitegravir, Dutasteride, and Oxybutynin attenuated the NMDA-induced breakdown of the mitochondrial membrane potential. Patch clamp electrophysiological recordings in NMDA receptor-expressing HEK293 cell lines and primary mouse hippocampal neurons revealed that Elvitegravir does not act at the NMDA receptor and does not affect the function of glutamatergic synapses. In summary, we have developed a cost-effective and easy-to-implement screening assay in primary neurons and identified Elvitegravir as a neuro- and mitoprotective drug that acts downstream of the NMDA receptor.

## Introduction

Glutamate toxicity is a basic pathomechanism that contributes to neuronal cell death in a wide range of acute and chronic neurodegenerative and neuroinflammatory diseases. This includes ischemic stroke, traumatic brain injury, Alzheimer’s disease, Huntington’s disease, Amyotrophic Lateral Sclerosis, and Multiple Sclerosis [1–4]. During acute trauma and chronic disease progression, excess glutamate is released from dysfunctional or dying neurons and/or from glial cells or activated immune cells. Under pathological conditions, binding of glutamate to the *N*-methyl-D-aspartate (NMDA) receptor on extrasynaptic neuronal membranes triggers signaling cascades that lead to mitochondrial permeability transition. This process is characterized by a breakdown of the mitochondrial membrane potential, excessive generation of reactive oxygen species, loss of ATP generation, and release of pro-apoptotic factors [5–7]. Accordingly, loss of mitochondrial structure and function is a key step in glutamate-mediated neuronal cell death [3, 8].

Despite its fundamental role in many neurodegenerative diseases, to date few clinically approved drugs are available that target glutamate toxicity. This is due—at least in part—to the fact that NMDA receptors have essential functions in neuronal physiology and thus are not well suited as drug targets [9–11]. We therefore aimed to identify novel compounds that act downstream of toxic NMDA receptor signaling and can protect mitochondria from glutamate toxicity. NMDA receptor signaling, however, is complex and its outcomes may differ based on NMDA receptor subunit composition, which is developmentally regulated. In addition, neuronal NMDA receptors are differentially localized to specialized subcellular compartments such as postsynaptic versus extrasynaptic membranes. Synaptic NMDA receptors promote physiological responses and cell survival, whereas extrasynaptic NMDA receptors promote cell death, possibly via coupling to specific second messenger cascades [9, 12–14]. This molecular and cell biological complexity is difficult, if not impossible, to recapitulate in cell lines. To overcome this limitation, we developed an automated microscopy-based screening assay in primary mouse cortical neurons. As a proof of principle, we screened a commercially available library of 424 FDA-approved drugs. Using our assay, we confirmed several known neuroprotective drugs and identified the anti-retroviral drug Elvitegravir as a neuro- and mitoprotective compound.

## Materials and methods

### Animals

C57BL/6NCrl mice (Charles River) were used in this study. Animals were maintained in pathogen-free and light- (12 h light/12 h dark) and temperature-controlled (22 °C ± 2 °C) conditions. Food (LasVendi Rod 16 or Rod 18) and water were available ad libitum*.* Animals were housed in conventional cages with ABBEDD LT-E-001 bedding material. Animal welfare was assessed daily by staff of the animal facility. All procedures were carried out in accordance with German guidelines for the care and use of laboratory animals and with the European Community Council Directive 2010/63/EU, and had full Home Office ethical approval (University of Heidelberg Animal Welfare Office and Regierungspraesidium Karlsruhe).

### Preparation of primary cortical neurons

Cortical neurons from newborn C57BL/6NCrl mice of both sexes were prepared and maintained as described previously [15]. In brief, 40,000 cells per well were seeded into Poly-D-Lysine coated 96-well plates (BD Biocoat 356,640) and were grown in Neurobasal-A medium (Life Technologies) supplemented with B27 (Life Technologies), 0.5 mM glutamine, and 1% rat serum. To prevent the proliferation of glial cells, cytosine β-D-arabinofuranoside (Sigma-Aldrich, 2.8 μM) was added on day in vitro (DIV) 3. On DIV 8 growth medium was exchanged to a defined minimal medium consisting of a mixture of buffered salt-glucose-glycine (SGG) solution [10 mM Hepes (pH 7.4), 114 mM NaCl, 26.1 mM NaHCO_3_, 5.3 mM KCl, 1 mM MgCl_2_, 2 mM CaCl_2_, 30 mM glucose, 1 mM glycine, 0.5 mM sodium pyruvate, and 0.001% phenol red] and phosphate-free Eagle’s minimum essential medium (MEM, Life Technologies) (9:1 vol:vol), supplemented with insulin (7.5 μg/ml), transferrin (7.5 μg/mI), and sodium selenite (7.5 ng/ml) (ITS supplement, Sigma-Aldrich). Experiments were performed after a culturing period of 10–12 DIV.

### Preparation of primary hippocampal neurons

Primary mouse hippocampal neurons were prepared and maintained as previously described [15]. Neurons were plated onto 12 mm diameter glass coverslips. All patch clamp recordings were performed after a culturing period of 14 to 18 DIV during which hippocampal neurons expressing markers for either glutamate (~ 90% of neurons) or GABA (~ 10% of neurons) develop a rich network of processes, express functional NMDA-type and 2-amino-3-(3-hydroxy-5-methyl-isoxazol-4-yl) propanoic acid (AMPA)/kainate-type glutamate receptors, and form synaptic contacts [16]. Viral transduction of neurons with the channelrhodopsin-2 (ChRII) mutant T159C [17] combined with an mCherry marker took place on DIV 8.

### Small molecule libraries

A small molecule library containing 424 FDA approved compounds provided as 10 mM stock solutions in DMSO, and a natural product library containing 146 compounds provided as 10 mM stock solutions in DMSO were purchased from SelleckChem. See Tables S1 and S2 for details.

### Drug treatment for cell death assay

On DIV 10, compounds were applied to the cells reaching an end concentration of 10 μM. Cells were incubated for 30 min at 37 °C, 5% CO_2_ before adding NMDA (30 μM). Cells were incubated for another 10 min in the incubator, then washed twice with defined minimal medium containing 10 μM of the respective compound and incubated for an additional 20 h before readout. Untreated (no NMDA) and NMDA-only stimulated wells were treated with 0.1% DMSO.

### Cell death assay

Twenty hours after NMDA treatment, cells were fixed and stained by direct addition of a mixture of formaldehyde (4% final concentration) and Hoechst 33258 (2 μg/ml final concentration). After 15 min of incubation at room temperature, cells were washed twice with phosphate buffered saline. Subsequently, 12 images per well were recorded with a 12-bit camera (Hamamatsu 480,674) attached to an automated fluorescence microscope (Olympus IX81, objective UPlanSApo 10x, NA: 0.4, DAPI filter set) at the Advanced Biological Screening Facility, BioQuant, Heidelberg. Dead neurons were identified by amorphous or shrunken nuclei using CellProfiler and CellProfiler Analyst software [18]. For all cell death analyses the experimental unit (N) is defined as an independent experiment using an independent neuronal preparation. For each independent experiment, data from 3 replicate wells (i.e., from 36 images), representing an average of 3000 cells was analyzed per condition. The survival rate S was calculated by dividing the number of living cells by the total number of cells. The survival rate was then used to calculate the protection score according to $\frac{S(compound)-S(vehicle)}{S(untreated)-S(vehicle)}\times 100=\% protection$, where ‘compound’ refers to compound + NMDA treatment, ‘vehicle’ refers to vehicle + NMDA treatment, and ‘untreated’ refers to DMSO control. Some compounds exhibited strong neurotoxic effects that resulted in low total numbers of cells. This precluded a reliable calculation of a protection score. Therefore, protection scores were not determined for compounds that resulted in a total cell count of less than 10% of the average cell count from all screening experiments (see Table S2). In addition, protection scores could not be calculated for the compounds Mitoxantrone and Cerubidine (Daunorubicin) because their color interfered with the fluorescence-based assay.

### Measurement of mitochondrial membrane potential changes

Live imaging was performed in 96-well plates (BD Biocoat 356,640) on an automated fluorescence microscope (Olympus IX81, objective UPlanSApo 10x, NA: 0.4) at the Advanced Biological Screening Facility, BioQuant, Heidelberg. Cells were incubated for at least 2 h with 50 nM tetramethylrhodamine ethyl ester (TMRE) in 100 μl imaging buffer (SGG without bicarbonate and phenol red) per well prior to adding compounds (10 μM). After 20 min of compound incubation, cells were stimulated with 30 μM NMDA. Time-lapse imaging commenced immediately after NMDA application. Due to initial autofocus determination, acquiring the first image of all wells took 124 s for each 96-well plate. Subsequently, one field of view per well in each of 25 wells was imaged in parallel for 20 min at a rate of one image every 30 s. 3–6 replicate wells per compound and 5–10 replicate wells of untreated and NMDA only-treated cells were imaged in each independent experiment. Fluorescence intensity was measured using FIJI with the StackReg plugin [19, 20]. In short, images of each well were aligned and background corrected, and fluorescence intensity was measured over time within automatic threshold-based regions of interest. Loss of TMRE signal was quantified by determining the area under the curve with Prism software (GraphPad).

### Automated patch clamp

HEK293 cell lines stably expressing human GluN1 (UniProt ID Q05586)/GluN2A (Uniprot Q12879) (CT6120, ChanTest Cell Lines, Charles river) or human GluN1/GluN2B (Uniprot Q13224) (CTN6121, ChanTest Cell Lines, Charles River) NMDA receptors were maintained in Dulbecco’s Modified Eagle’s medium (DMEM) supplemented with 10% FBS, sodium pyruvate, non-essential amino acids, and 0.5% penicillin/streptomycin at 37 °C and 5% CO_2_. Expression of GluN1, GluN2A, and GluN2B was induced by tetracycline. Voltage clamp recordings were performed using the Sophion Qube platform which performs 384 parallel and independent patch-clamp recordings with digitally controlled microfluidics (Tcan D300) on a disposable, 10-hole QChip. Data was filtered for quality control thresholds for seal resistance and holding current. The following extracellular solution was used (in mM): NaCl, 145; KCl, 4; HEPES, 10; Glucose, 10; CaCl_2_, 2; pH 7.4. The following intracellular solution was used (in mM): CsF, 70; CsCl, 70; HEPES, 10; EGTA, 1; 316 mOsm, pH 7.2. IC_50_ estimates for Elvitegravir and NMDA receptor antagonists were generated at −70 mV in Mg^2+^-free extracellular solution against responses to glycine (100 μM) plus NMDA (90 μM for GluN1/GluN2A and 40 μM for GluN1/GluN2B). These concentrations represent saturating glycine and EC_80_ NMDA concentrations for glutamate receptor activation as determined a priori for each cell line.

### Whole-cell patch clamp recordings

Whole-cell patch clamp recordings were made at 32 °C from DIV14–18 primary hippocampal neurons plated on coverslips secured with a platinum ring in a recording chamber (Open access chamber-1, Science Products GmbH, Hofheim, Germany) mounted on a fixed-stage upright microscope (BX51WI, Olympus) with heated in-line perfusion (TC324B, Warner Instruments Corporation) running constantly at 2–3 ml per minute. Recordings were made with a Multiclamp 700B amplifier, digitized through a Digidata 1322A A/D converter and quantified using pClamp 10 software (Molecular Devices, CA, USA). Access resistance (range: 8–20 MΩ) was monitored regularly during voltage clamp recordings and data was rejected if changes greater than 20% occurred. Patch electrodes (3–5 MΩ) were made from borosilicate glass (1.5 mm, WPI, Sarasota, FL, USA). The intracellular solution was composed of (in mM): cesium gluconate (90), HEPES (10), EGTA (5), CaCl_2_ (0.5), tetraethylammonium (10), QX-314 (4), ATP (4), GTP (0.5), K_2_-phophocreatine (10). The extracellular solution was composed of (in mM): NaCl (125), KCl (3.5); MgCl_2_ (1.3), NaH_2_PO_4_ (1.2), CaCl_2_ (2.4), glucose (10), NaHCO_3_ (21); equilibrated with 95% O_2_ and 5% CO_2_; pH 7.35; 325 mOsm. Where indicated, MgCl_2_ was increased to 4 mM to stabilize the membrane potential and suppress recurrent EPSC generation. For recordings of AMPA receptor-mediated EPSCs, the NMDA receptor blocker, dizocilpine maleate (MK-801, 10 μM), and the GABA_A_ receptor blocker, SR 95531 hydrobromide (gabazine, 2 μM), were added to the extracellular solution. For recordings of NMDA receptor-mediated EPSCs, the AMPA receptor blocker, 2,3-Dioxo-6-nitro-1,2,3,4-tetrahydrobenzo [f]quinoxaline-7-sulfonamide disodium salt (NBQX, 5 μM), gabazine (2 µM), and the NMDA receptor coagonist, glycine (100 μM), were added to the extracellular solution. AMPA and NMDA receptor-mediated EPSCs were verified by the addition of NBQX (5 μM) or D-(L)-2-Amino-5-phosphonopentanoic acid (DL-APV, 50 μM), respectively. ChRII is a light-gated cation channel that has been extracted from the green algae *Chlamydomonas reinhardtii*. Fluorescent excitation light was generated by a 470 nm LED for ChRII excitation and by a 594 nm LED for mCherry (pE2, CoolLEDs, Andover, UK). Excitation light was passed through ET470/40 and HQ570/20 cleanup filters and emission light was passed through ET585 long-pass dichroic and ET620/60 emission filters (AHF Analysetechnik, Tuebingen, Germany). Four hundred-seventy nm LED triggering was controlled directly from the digitizer.

### Statistical analyses

Statistical analyses were performed with Estimation Statistics Beta [21], Prism (GraphPad) or OriginPro (OriginLab). To determine bias-corrected and accelerated confidence intervals in Estimation Statistics Beta, 5000 bootstrap samples were taken. All IC_50_ or EC_50_ values were generated using a logistic fit to the Hill equation. Experimental units (N) for each experiment are defined in the respective methods sections and in the figure legends.

### Illustrations

Graphs were generated with Prism and Estimation Statistics Beta [21]. Heatmaps were generated with Heatmapper [22]. Figures were assembled with Adobe Photoshop and Adobe Illustrator.

## Results

### Development of a robust cell death screening assay in primary neurons

**Fig. 1:**
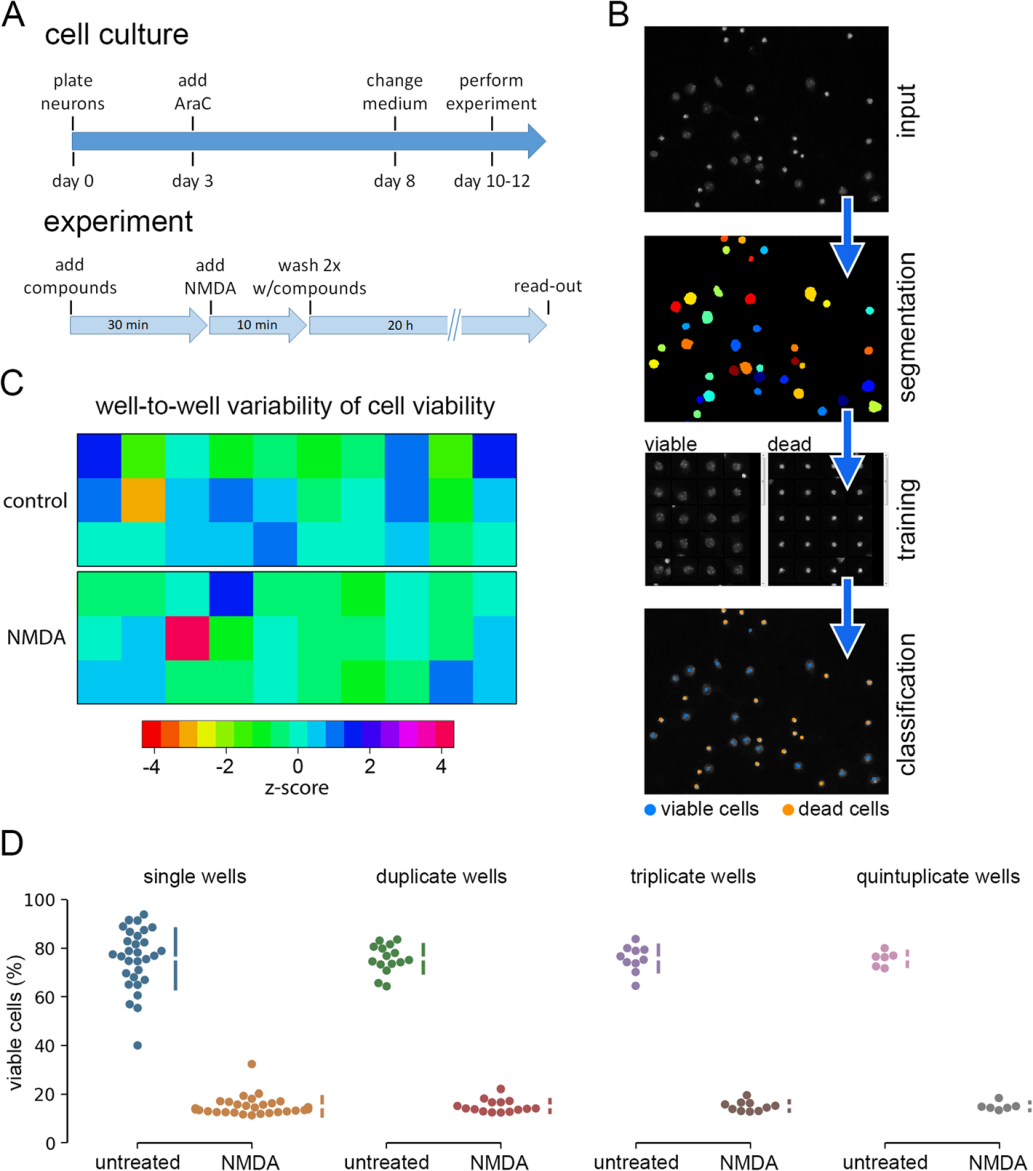
Development of a screening assay using primary neurons. **a** Schematic representation of the workflow. **b** Example images illustrating data analysis with CellProfiler (segmentation) and CellProfiler Analyst (training and classification). Dead neurons can be identified based on their shrunken and brightly stained nuclei. **c** Heatmap illustrating the variability of neuronal viability between technical replicate wells. Z-scores (the number of standard deviations by which each value differs from the mean) were calculated independently for control and NMDA-treated wells. **d** Comparison of cell viability values that were either calculated for each single well of the plate shown in **c***,* or based on the summed cell counts from groups of two, three, or five wells from that plate. Error bars indicate the SD

To analyze excitotoxic cell death, primary cortical neurons were treated with 30 μM NMDA for 10 min followed by two washes with fresh medium, and cell death was assessed after 20 h (Fig. 1a). This protocol has previously been shown to robustly induce neuronal cell death that can be attenuated by various pharmacological pre-treatments or genetic manipulations [15, 23, 24].

When choosing a simple and cost-effective assay to quantify cell death in cultured primary neurons, we took advantage of the fact that necrotic neurons can be readily identified by the distinct morphology of their nuclei and thus be distinguished from live cells [15, 23, 25]. Therefore, we stained fixed neurons with the nuclear dye Hoechst 33258 and used the CellProfiler Analyst tool [18] to classify cells as live or dead (Fig. 1b). Quantifying the number of both dead and live cells within each well provides an internal normalization for the total number of cells per analysis region. This reduces the variability of the assay without the need for additional assays such as quantification of total protein amount per well that is typically used in enzyme-based cell viability assays.

We initially performed cell death assays using primary neurons grown in 24-well, 96-well, and 384-well plates and found that the 96-well format provided the best trade-off between sample throughput, operability, and cell viability. Because primary neurons are highly sensitive to osmotic stress that results from media evaporation [26], we did not seed neurons into the outermost wells of the 96-well plates, which are affected most by evaporation.

To further benchmark our assay, we assessed the well-to-well variability of cell viability, which we expected to be larger in primary neurons than in the cell lines typically used in viability analyses. In an experiment in which 30 wells in a 96-well plate were treated with NMDA and 30 wells were left untreated, NMDA robustly reduced survival, whereas cell survival in individual untreated wells was rather variable (Fig. 1c, d). Viability in untreated wells ranged from 40 to 94% with a mean of 75.7% and a standard deviation of 12.27%. Calculating viability based on summed cell counts from two, three, or five replicate wells reduced the standard deviation to 5.8, 5.48%, or 3.07%, respectively (Fig. 1d). Therefore, as a trade-off between robustness and throughput, we decided to test each compound in triplicate wells in our proof-of-principle screens, such that each data point was derived from the summed cell counts in three wells.

### Screening of small molecule libraries

**Fig. 2:**
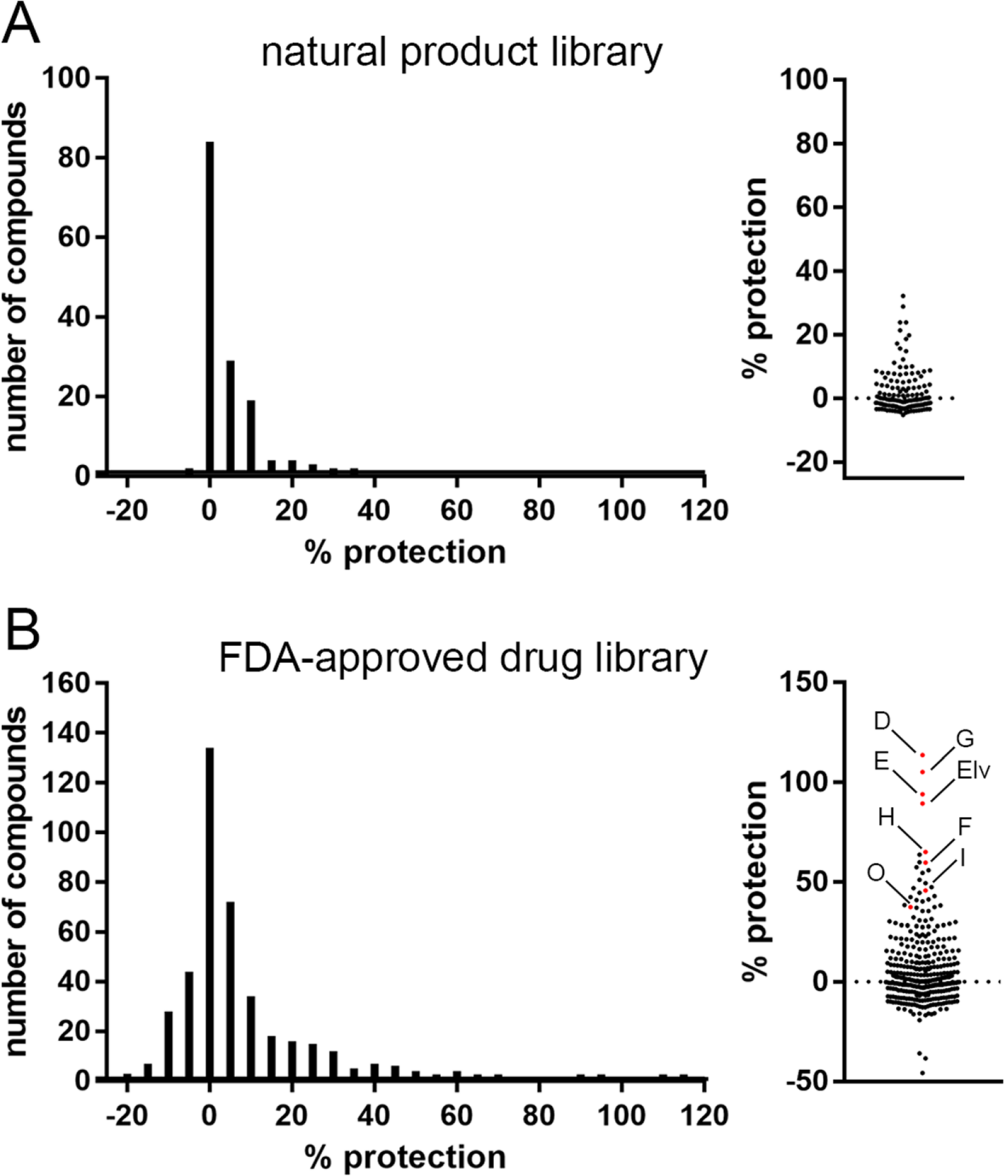
Summary data of protection scores that were obtained in the small molecule screens. **a-b** Binned histogram (left) and scatter plot (right) of protection scores from compounds in the natural product library **a** and the library of FDA-approved drugs **b**. The five most protective compounds and compounds that were studied in follow-up experiments are indicated by red dots in the scatter plot and are labelled as D = Dutasteride, G = Grape seed extract, E = Eltrombopag, Elv = Elvitegravir, H = Haloperidol, F = Finasteride, I = Isoniazid, O = Oyxybutynin

As a proof of principle, we next used our assay to screen two commercially available drug libraries. In these screens, primary cortical cells were incubated with test compounds at 10 μM concentration for 30 min before addition of 30 μM NMDA. After 10 min NMDA treatment, cells were washed with fresh medium and kept in the presence of test compounds for 20 h until assessment of cell viability. First, we screened a library of 146 structurally diverse natural products (Fig. 2a, Table S1). In this screen, protection scores ranged from − 5.3% (slightly toxic) to 32.2% (moderately protective). We then screened a library of 424 FDA-approved drugs. This library covers structurally diverse drugs from several fields such as oncology, cardiology, immunology, and neurology/psychiatry. We found a number of compounds that exacerbated NMDA-induced cell death (negative protection scores), a majority of compounds that exhibited little to no effect on cell death, and 12 compounds that conferred > 50% protection from NMDA-induced cell death (Fig. 2b, Table S2). The latter include known neuroprotective compounds such as grape seed extract, Haloperidol, Enalapril (Vasotec), Finasteride, and Dutasteride, as well as novel candidates such as Elvitegravir.

### Characterization of selected hit compounds

**Fig. 3:**
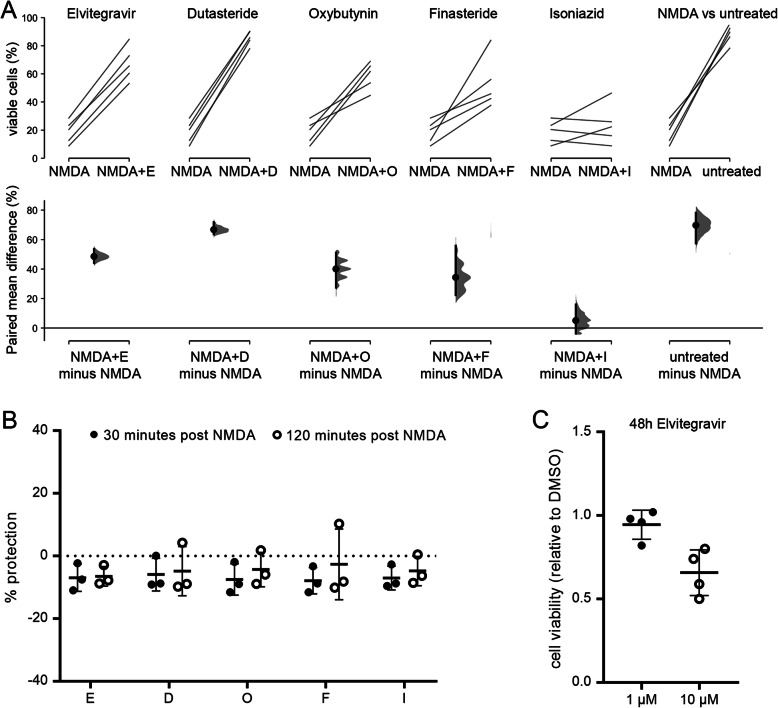
Verification and further characterization of selected compounds. **a** Neuroprotective properties of selected compounds were verified in independent NMDA toxicity assays using the same method as for the initial screen. The raw data on the upper axes depicts cell viability in NMDA-treated cells (shared among all comparisons) versus cells that were treated with NMDA plus the indicated compounds. A comparison between NMDA-treated and untreated cells is shown as a reference on the right. Each paired set of observations is connected by a line and represents the mean of an individual experiment. On the lower axes, each paired mean difference is plotted as a bootstrap sampling distribution. Mean differences are depicted as dots; 95% confidence intervals are indicated by the vertical error bars. The *p* values of the two-sided permutation *t*-tests are: NMDA vs NMDA + E, 0.000; NMDA vs NMDA + D, 0.000; NMDA vs NMDA + O, 0.000; NMDA vs NMDA + F, 0.067; NMDA vs NMDA + I, 0.448; NMDA vs untreated, 0.000. For each permutation *p* value, 5000 reshuffles of the control and test labels were performed. **b** Neurons were treated with 30 μM NMDA for 10 min and the indicated compounds were added 30 or 120 min after washout of NMDA. Protection scores were determined after 20 h. Round symbols represent the mean of each independent experiment, horizontal lines represent the mean of all experiments, error bars indicate the SD. **c** Neurons were treated for 48 h with 0.1% DMSO, 1 μM Elvitegravir, or 10 μM Elvitegravir and results were normalized to values from DMSO-treated controls. Round symbols represent the mean of each independent experiment, horizontal lines represent the mean of all experiments, error bars indicate the SD. Abbreviations are: untreated, no NMDA; D, Dutasteride; E, Elvitegravir; F, Finasteride; I, Isoniazid; O, Oxybutynin

**Fig. 4:**
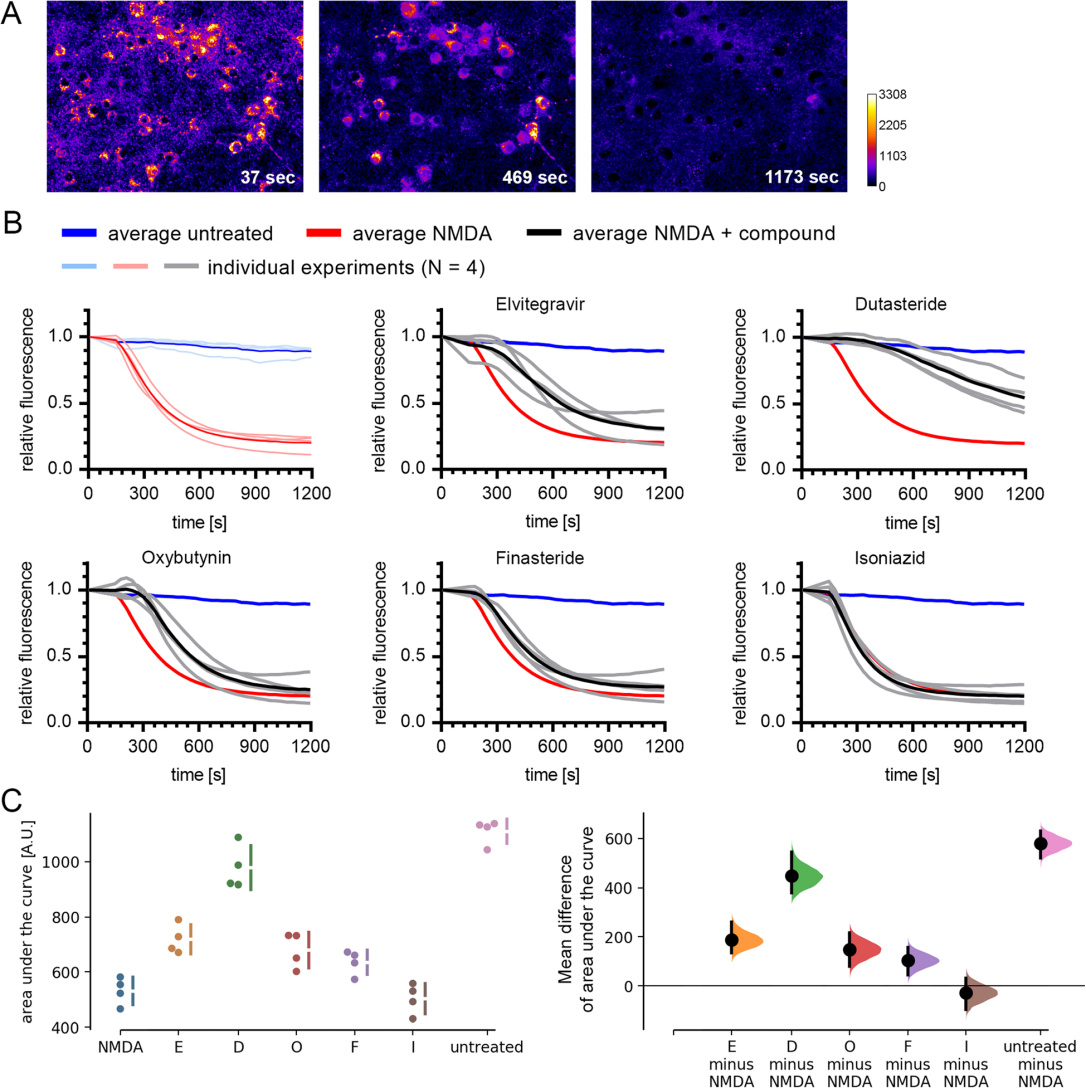
Assay for protection against NMDA-induced mitochondrial membrane potential breakdown. **a** Three images from a TMRE time-lapse experiment, acquired at the indicated times after NMDA application. Colorization represents background-corrected TMRE intensity (see calibration bar). **b** Analysis of time lapse experiments. The upper left graph shows individual traces for untreated and NMDA-treated cells from four independent experiments (light colored traces depict the means of 3–10 replicate wells per experiment) and the mean from all experiments (bold traces). The remaining graphs show individual and mean traces for cells that were treated with compound + NMDA. The mean traces for untreated and NMDA-only treated cells are included in each graph for comparison. TMRE intensity was normalized to that of the first image which was taken immediately after NMDA application (time = 0). **c** Quantification of NMDA-induced loss of TMRE fluorescence. On the left graph, dots depict the mean of each individual experiment and vertical error bars indicate the SD of all experiments. On the right graph, mean differences are plotted as bootstrap sampling distributions. Each mean difference is depicted as a dot. Each 95% confidence interval is indicated by the vertical error bars. The *p* values of the two-sided permutation *t*-tests are: E, 0.0226; D, 0.006; O, 0.0006; F, 0.0376; I, 0.487; untreated, 0.006. For each permutation *p* value, 5000 reshuffles of the control and test labels were performed


We selected five neuroprotective compounds from our screen for further characterization. First, we confirmed their neuroprotective effect in independent experiments using the same method as for the initial screen (Fig. 3a). We next assessed if these compounds confer neuroprotection when applied after the NMDA insult. Such a post-insult protective effect would be desirable for potential clinical applications. Application of the compounds at 30 min or 2 h after NMDA washout, however, did not attenuate neuronal cell death (Fig. 3b). Following up on a recent study that reported toxic effects of Elvitegravir in primary rat cortical neurons [27], we assessed the effect of 48 h Elvitegravir treatment on our mouse cortical neurons. Similar to this previous study, we observed an ~ 34% decrease of viable cells after 48 h treatment with 10 μM Elvitegravir (Fig. 3c). Finally, we asked if the compounds are able to attenuate NMDA-induced mitochondrial membrane potential breakdown, a key event in excitotoxic cell death [3]. We used the cationic dye TMRE to visualize the mitochondrial membrane potential and its NMDA-induced breakdown in live cells [28, 29]. To facilitate the analysis of all five compounds in parallel, TMRE live imaging was performed in 96-well plates on an automated microscope (Fig. 4a, supplementary Movie 1). In untreated cells, TMRE fluorescence remained constant throughout the 20-min observation time. In contrast, application of 30 μM NMDA resulted in rapid loss of mitochondrial TMRE fluorescence, indicative of mitochondrial depolarization (Fig. 4b, supplementary Movie 1). Pre-treatment with Elvitegravir, Dutasteride, or Oxybutynin resulted in delayed and less pronounced loss of TMRE fluorescence upon NMDA treatment, revealing mitoprotective properties of these compounds (Fig. 4b, c).

**Additional file 3: Supplementary Movie 1.** 20-min time-lapse imaging of primary cortical neurons stained with TMRE following NMDA (30 μM) insult in the presence or absence of 10 μM protective compound. Image stacks were registered and have equivalent contrast to enable comparison.

### Pharmacological characterization of Elvitegravir at NMDA receptors

**Fig. 5:**
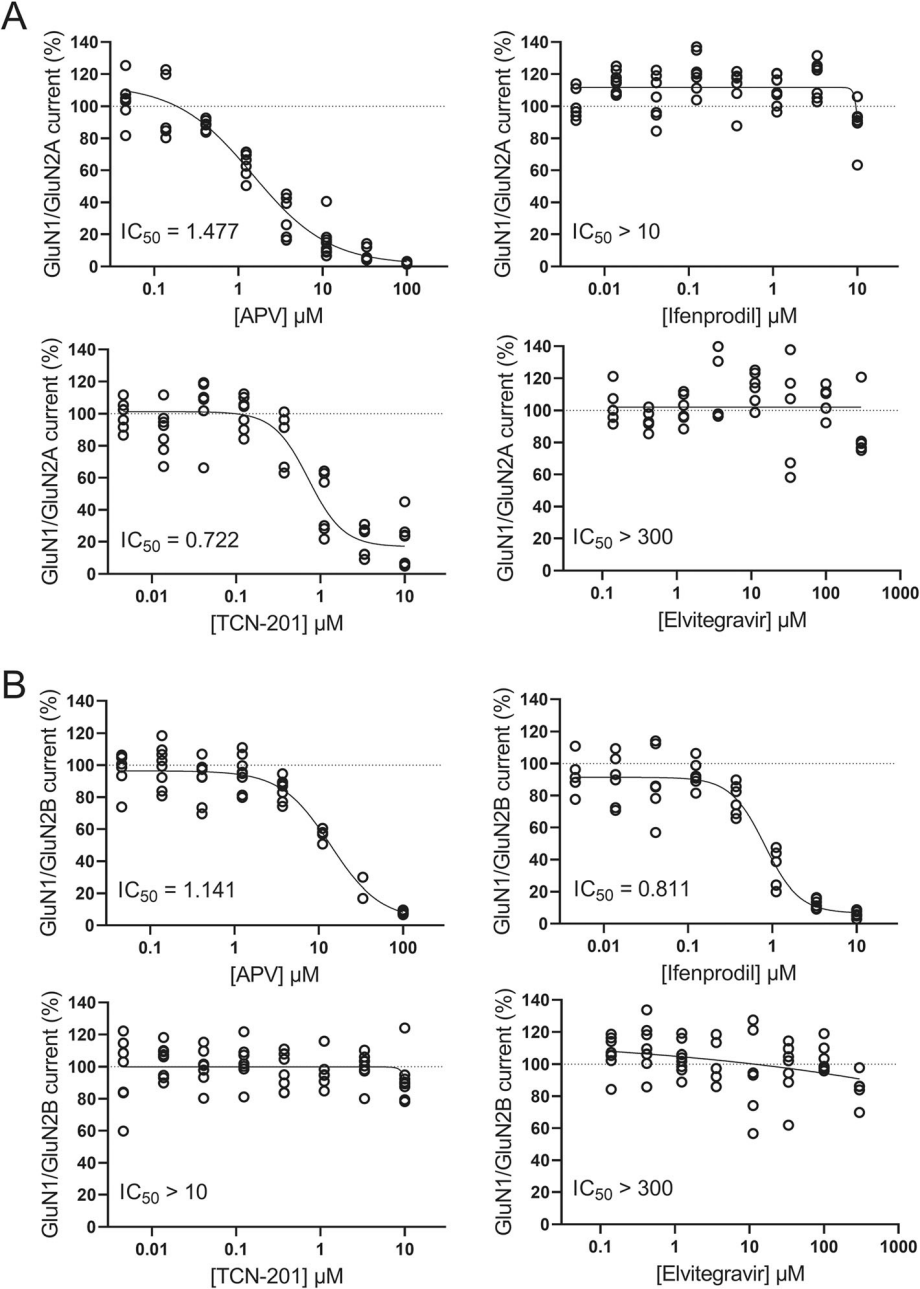
Elvitegravir does not affect NMDA receptor function in HEK293 cells. Automated patch clamp recordings were performed using the Sophion Qube platform to generate antagonistic dose-response curves of Elvitegravir and NMDA receptor antagonists, D-APV, Ifenprodil, and TCN-201 for human GluN1/GluN2A **a** or GluN1/GluN2B **b** NMDA receptors expressed in HEK293 cell lines. Responses to NMDA and glycine (see methods section) were recorded at −70 mV in Mg^2+^-free extracellular solutions and normalized to responses of the same cells recorded in the absence of Elvitegravir or antagonists. GluN1/GluN2A was blocked by the non-selective NMDA receptor antagonist, D-APV (IC_50_: 1.477 μM) and the selective GluN2A blocker, TCN-201 (IC_50_: 0.722 μM) but not the GluN2B antagonist, Ifenprodil (IC_50_ > 10 μM) or Elvitegravir (IC_50_ > 300 μM). GluN1/GluN2B was blocked by the non-selective NMDA receptor antagonist, D-APV (IC_50_: 1.141 μM) and the selective GluN2B blocker, Ifenprodil (IC_50_: 0.811 μM) but not the GluN2A antagonist, TCN-201 (IC_50_ > 10 μM) or Elvitegravir (IC_50_ > 300 μM). Data are shown as individual cell values and a logistic fit of the Hill equation to the data from which IC_50_ values were estimated. *N* = 2–8 cells from two independent experiments

Since Elvitegravir has not previously been described as neuroprotective, we focused on that compound and aimed to elucidate its neuroprotective mechanism. Inhibition of NMDA receptors by Elvitegravir would provide a straightforward explanation for its neuroprotective properties. As discussed above, however, the NMDA receptor is not well-suited as a drug target. To help evaluate the clinical potential of Elvitegravir, we thus tested if this compound acts directly at NMDA receptors rather than at a downstream target. To assess the IC_50_ of Elvitegravir for NMDA receptor-mediated currents we employed a high throughput automated patch clamp system to measure NMDA receptor currents in HEK293 cells stably expressing GluN1/GluN2A or GluN1/GluN2B NMDA receptors. Currents were measured at −70 mV in Mg^2+^-free solutions. Analysis found that Elvitegravir had no effects on NMDA-evoked currents for either GluN1/GluN2A or GluN1/GluN2B receptors (IC_50_ > 300 μM) (Fig. 5). Thus Elvitegravir’s protection against NMDA-induced toxicity is not mediated by direct antagonism of NMDA receptors.

### Measuring optogenetically evoked AMPA and NMDA receptor-mediated synaptic currents with patch clamp recordings

In any potential in vivo application, compounds that protect against glutamate-induced excitotoxicity should not interfere with glutamatergic synaptic transmission, which is critical for the plasticity underlying learning and memory. To assess the effects of Elvitegravir on synaptic glutamate receptor function in a neuron, we performed whole-cell patch clamp recordings in primary hippocampal cultures (DIV 14–16) from mice. AMPA and NMDA receptor-mediated synaptic contacts in such cultures have previously been shown to mediate network activity whose NMDA receptor-dependent modulation serves as an in vitro model for synaptic plasticity [16]. Synaptic AMPA and NMDA receptor function was assessed from single and paired pulse EPSC events evoked via the optogenetic stimulation of action potentials in presynaptic neurons expressing ChRII. This optogenetic method was chosen over traditional electrical stimulation due to difficulties with the latter technique in achieving stable recordings of single EPSC responses over prolonged recordings in monolayer cultures, especially in the presence of GABA_A_ receptor antagonists, which promote epileptiform activity.

### Verification of the stability of ChRII responses

**Fig. 6:**
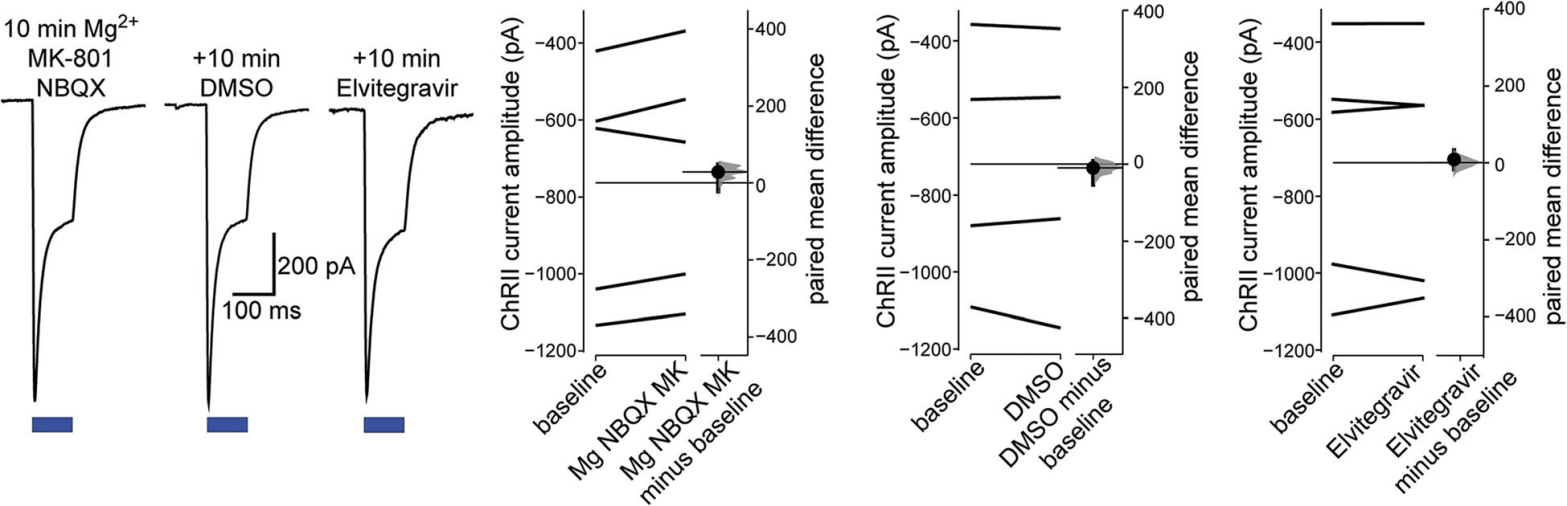
Characterization and validation of ChRII activation with light. On the left are representative whole-cell patch clamp recordings (Vhold = −70 mV) from an mCherry-positive neuron in a DIV 16 primary hippocampal culture infected with rAAVs encoding for mCherry and the channelrhodopsin-2 (ChRII) mutant T159C on DIV 8. Recordings were made from the same cell after 10 min sequential application of blockers (Mg^2+^, 4 mM; NBQX, 5 μM; MK-801, 10 μM), vehicle (DMSO 0.1%) and Elvitegravir (20 μM). One-hundred ms blue (470 ± 20 nm) light pulses (blue bars) evoke downward inward current deflections with prominent inactivation over the 100 ms pulse duration. The raw response amplitude data for all cells (*N* = 4 or 5) is plotted on the right as paired observations connected by a line and the paired mean difference is plotted as a bootstrap distribution. Mean differences are depicted as dots with the 95% confidence intervals indicated by the vertical error bars. The *p* values of the two-sided permutation *t*-tests are: baseline vs Mg/NBQX/MK-801, 0.125; baseline vs DMSO, 0.757; baseline vs Elvitegravir, 0.747

To investigate the effects of Elvitegravir on synaptic transmission we used light activation of ChRII to evoke action potentials in ChRII and mCherry co-expressing presynaptic neurons for the generation of EPSCs in postsynaptic ChRII-negative cells. To characterize, optimize, and verify the stability of our method, we first performed patch clamp recordings from mCherry-positive neurons. These neurons exhibited light-activated currents (273 pA to 1103 pA) in 17 out of 17 cells, confirming co-infection with the ChRII-encoding recombinant adeno-associated virus (rAAV). These currents showed the expected rapid inactivation over a 100 ms light pulse which was unaffected by the application of either DMSO (0.1%) or Elvitegravir (20 μM dissolved in DMSO to a final concentration of 0.1%) (Fig. 6). Recovery from inactivation was apparent within 30 s in accordance with the published recovery time (τ_rec_) of 16 s [17]. These observations suggest that ChRII is not affected by Elvitegravir.

### The effect of Elvitegravir on synaptic AMPA and NMDA receptor function

**Fig. 7:**
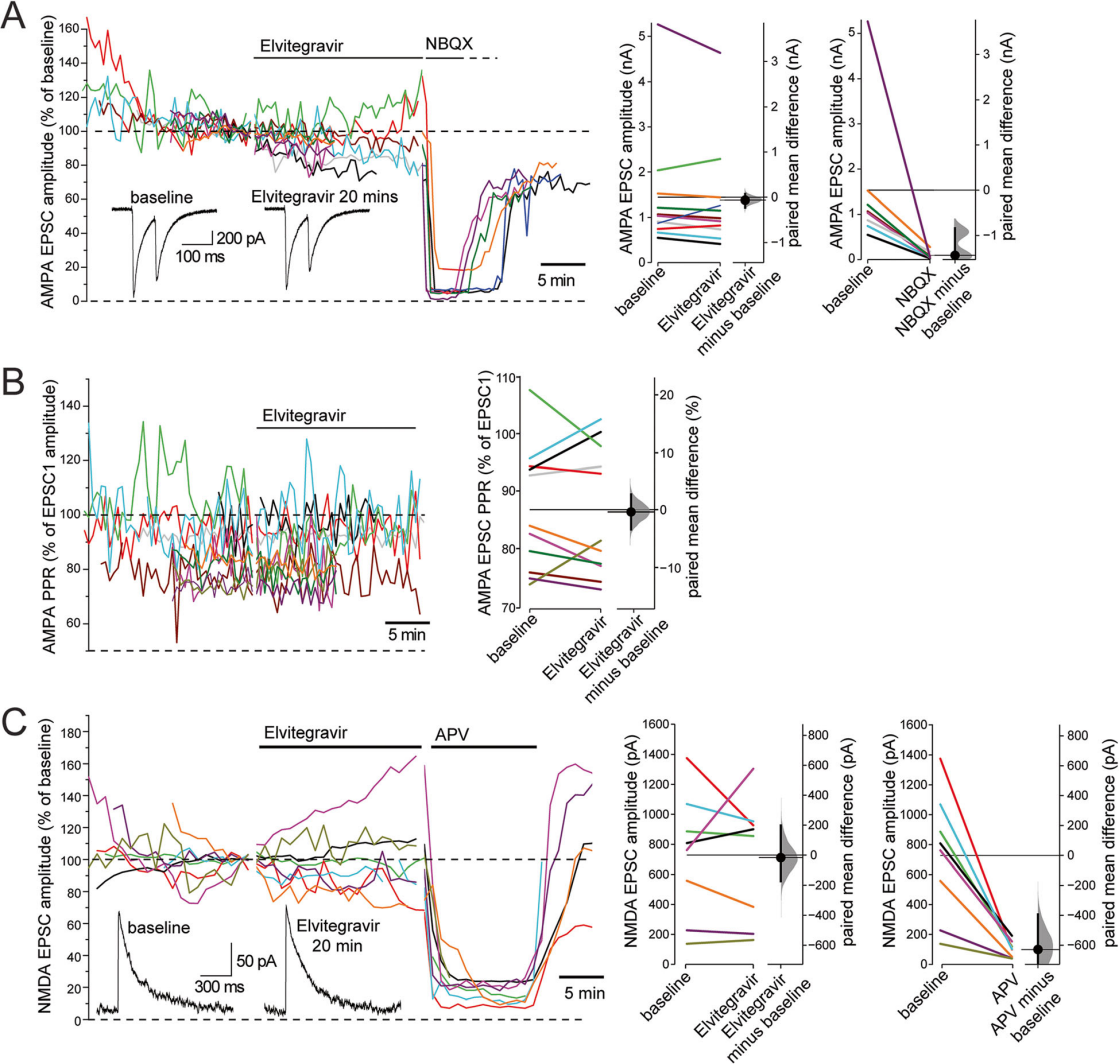
Elvitegravir does not affect glutamate receptor-mediated EPSCs or glutamate release probability in hippocampal neurons. Whole-cell recordings from mCherry- and ChRII-negative cells of postsynaptic AMPA **a, b** and NMDA **c** receptor-mediated EPSCs evoked with 0.7 ms light pulses in DIV 14–18 primary hipppocampal cultures. Traces in left panels show responses over the timecourse of experiments. Plots on the right show the data for all cells as paired observations connected by a line as well as the paired mean difference, plotted as a bootstrap distribution. Mean differences are depicted as dots with the 95% confidence intervals indicated by the vertical error bars. **a** The amplitude of the first AMPA EPSC response to a pair (100 ms interstimulus interval) of light pulses was unaffected by Elvitegravir (20 μM) application for 10 min (*N* = 11 cells, *p* = 0.469) or 20 min (*N* = 5 cells, *p* = 0.874). EPSCs were rapidly blocked by NBQX (5 μM) in a subset of cells (*N* = 8; *p* < 0.005). Insets show examples of paired EPSC recordings. **b** The paired pulse ratio (PPR) was quantified as the ratio of the amplitude of EPSC2 / EPSC1 and was not affected by Elvitegravir application for 10 min (*N* = 11 cells, *p* = 0.469) or 20 min (*N* = 5 cells, *p* = 0.260). **c** NMDA receptor-mediated EPSCs were recorded at +40 mV in a different set of neurons. Elvitegravir application for 20 min did not affect the amplitude of responses (*N* = 8 cells, *p* = 0.822), which were rapidly blocked by DL-APV (*N* = 8 cells, *p* < 0.005, two-sided permutation *t*-test)

AMPA receptor-mediated EPSCs were recorded from mCherry- and ChRII-negative cells and pharmacologically isolated with GABA_A_ and NMDA receptor blockers. In addition, to suppresses recurrent activity, which is promoted by GABA_A_ blockers, we increased the extracellular Mg^2+^ concentration to 4 mM. The DMSO used to dissolve Elvitegravir was added during baseline recordings to an equivalent of 0.01%. Under these conditions, AMPA receptor-mediated EPSCs recorded at a holding potential of −70 mV were evoked with 0.7 ms light pulses at 30 s intervals until stable response amplitudes were achieved. Further addition of Elvitegravir (20 μM) did not affect AMPA EPSC amplitudes over 10 to 20 min (Fig. 7a). Immediate and almost complete blockade, however, was achieved with the addition of the AMPA receptor blocker NBQX.

The ratio of responses to a pair of synaptic stimuli provides an indication of neurotransmitter release probability [30–32]. A relative decrease in the second response is indicative of an increased synaptic transmitter release probability [33]. This is typically quantified as a paired pulse ratio (PPR), calculated as the ratio of the second to the first postsynaptic response amplitude. To assess any impact of Elvitegravir on synaptic glutamate release probability, we used paired light pulses which produced a maximal PPR of around 0.9 using a 75 ms inter-pulse interval. Partial inactivation of ChRII reduces the second EPSC response amplitude and the magnitude of our PPRs in comparison to those from recordings using electrical stimulation [30–33]. Nonetheless, PPRs remained stable over prolonged recordings and were not affected by Elvitegravir application (Fig. 7b).

Finally, to assess the effect of Elvitegravir on synaptic NMDA receptor function we recorded NMDA receptor-mediated EPSCs at +40 mV in the presence of blockers of GABA_A_ and AMPA receptors as well as elevated Mg^2+^. Elvitegravir also showed no effect on the amplitude of NMDA receptor-mediated EPSCs over a 20 min period (Fig. 7c). Thus Elvitegravir blocked neither the presynaptic release probability of glutamate nor the postsynaptic AMPA and NMDA receptors that glutamate activates.

## Discussion

While pro-death signaling of extrasynaptic NMDA receptors is a common trigger of neuronal cell death in a wide range of neurodegenerative diseases, the NMDA receptor itself is not well-suited as a drug target [3, 9]. Therefore, to develop novel therapeutic strategies, it is important to identify compounds that act downstream of toxic NMDA receptor signaling. In this study, we developed a screening method to identify neuro- and mitoprotective compounds in mouse primary cortical neurons. This screening method is robust, scalable, and does not require specialized equipment or reagents. Compared to other commonly used methods that depend on enzymatic reactions to quantify cell death, such as quantification of lactate dehydrogenase (LDH) release or ATP levels, our microscopy-based screening method is highly cost-effective.

The hits that were identified in our screen include a number of previously described neuroprotective compounds. The antipsychotic drug Haloperidol has been described to protect striatal neurons against NMDA toxicity in vivo, most likely via interaction with the NMDA receptor [34, 35] but see [36]. The 5α-reductase inhibitors Finasteride and Dutasteride have been shown to protect against chemical ischemia and mitochondrial permeability transition in cultured neurons, most likely via modulation of voltage-gated potassium channels [37]. Enalapril (Vasotec) is an inhibitor of angiotensin converting enzyme and has been shown to protect against glutamate-mediated cell death in vitro and against focal cerebral ischemia in rats in vivo, via radical scavenging [38, 39]. These findings suggest that our screening method can reliably detect neuroprotective compounds that act via diverse targets and signaling mechanisms.

In our follow-up experiments we focused on the anti-retroviral compound Elvitegravir, as it has not previously been described as neuroprotective. Elvitegravir is an integrase inhibitor that is used to treat HIV infection. Its neuroprotective effect, however, cannot be explained by inhibition of viral integrase enzymes that are absent in naïve primary neurons. This is supported by our finding that the related integrase inhibitor Raltegravir was not neuroprotective in our screen and suggests an off-target effect being responsible for neuroprotection. Based on our electrophysiological characterization, its target is not the NMDA receptor. Thus, Elvitegravir exerts its neuroprotective effect by acting on a yet to be identified target that is located downstream of the NMDA receptor and upstream of mitochondrial membrane potential breakdown.

Administration of Elvitegravir following the excitotoxic insult failed to provide neuroprotection in our study. Thus, Elvitegravir is unlikely to be effective in treating acute insults like ischemic stroke or brain trauma. It might be effective, however, in the treatment of diseases that involve chronic glutamate toxicity, such as Alzheimer’s disease, Huntington’s disease, Amyotrophic Lateral Sclerosis, and Multiple Sclerosis. As an FDA-approved drug that has been used in a large number of patients for many years, the safety profile of Elvitegravir is well established. For a potential treatment of neurodegenerative diseases, however, considerable drug development appears necessary, including, e.g., an improvement of Elvitegravir’s blood-brain-barrier permeability [40, 41]. Toxic side-effects of prolonged Elvitegravir exposure are likely due to its activation of the integrated stress response (ISR) [27]. Further investigation of the mechanism of Elvitegravir’s off-target neurotoxic effect might identify strategies to attenuate its long-term toxicity. A promising example is the application of the small molecule ISR inhibitor, trans-ISRIB [27]. Alternatively, a combinatorial chemistry or structure-based computational drug screen approach might identify compounds similar to Elvitegravir without neurotoxic side-effects. To assess the applicability of improved Elvitegravir derivatives, their neuroprotective properties should be assessed in a range of neurotoxicity paradigms such as glutamate toxicity, oxygen-glucose deprivation, and oxidative stress. Although our in vitro electrophysiological .experiments did not provide evidence for an effect of Elvitegravir on glutamatergic synapses, the in vivo situation might be different. Given its low permeability through the blood-brain-barrier, potential neuroactive properties of Elvitegravir are unlikely to adversely affect the brain. This might be an issue, however, with modified compounds that have improved blood-brain-barrier permeability. Therefore, to assess the in vivo safety of improved Elvitegravir derivatives, potential effects on learning and memory should be tested. In recent years, several genetic and pharmacological strategies for mitoprotection have been shown to be effective in protecting against excitotoxic cell death in vitro and in vivo [23, 25, 42–46]. Our study identifies Elvitegravir as a novel, clinically approved compound for mitoprotective drug development and reports a robust, simple, and cost-effective screening method that might help identify additional drug candidates in the future.

## Supplementary information

**Additional file 1.** Table S1.

**Additional file 2.** Table S2.

## Data Availability

The datasets used and/or analyzed during the current study are available from the corresponding author on reasonable request.

## Abbreviations

AMPA: 2-amino-3-(3-hydroxy-5-methyl-isoxazol-4-yl) propanoic acid
APV: DL-2-amino-5-phosphonopentanoic acid
ChRII: Channelrhodopsin-2
DIV: Days in vitro
DMSO: Dimethylsulfoxide
EGTA: Ethylene glycol tetraacetic acid
EPSC: Excitatory post synaptic current
GABA: Gamma-aminobutyric acid
NBQX: 2,3-Dioxo-6-nitro-1,2,3,4-tetrahydr¬obenzo [f]quinoxaline-7-sulfonamide
NMDA: N-methyl-D-aspartate
rAAV: Recombinant adeno-associated virus
TMRE: Tetramethylrhodamine ethyl ester
